# Role of the gut microbiota and innate immunity in polycystic ovary syndrome: Current updates and future prospects

**DOI:** 10.1111/jcmm.18258

**Published:** 2024-03-28

**Authors:** Min Zhou, Jing Yu, Xuewei Li, Zheng Ruan, Shaohui Yu

**Affiliations:** ^1^ Department of Gynaecology The Affiliated Hospital to Changchun University of Chinese Medicine Changchun Jilin China; ^2^ Department of Endocrinology The Affiliated Hospital to Changchun University of Chinese Medicine Changchun Jilin China; ^3^ Department of Traditional Chinese Medicine 964th Hospital Changchun China

**Keywords:** gut microbiota, hormone, obesity, PCOS

## Abstract

Polycystic ovary syndrome (PCOS) is one of the modern intractable reproductive diseases. The female irregular menstruation, infertility, obesity, and so forth caused by PCOS have become a hot issue affecting family harmony and social development. The aetiology of PCOS is complex. In recent years, many scholars have found that its pathogenesis was related to the imbalance of gut microbiota. Gut microbiota can form two‐way communication with the brain through the ‘gut‐brain axis’ and affect the host's metabolism. Current research has confirmed that the gut microbiota can interfere with glucose and lipid metabolism, insulin sensitivity, hormone secretion and follicular development in women by altering intestinal mucosal permeability and secreting metabolites. In addition, the diversity and composition of gut microbiota of PCOS patients changed, which may affect the metabolic function of the gut microbiota and the ability to produce metabolites, and may also directly or indirectly affect the endocrine function. This study reviewed recent research advances about the role of gut microbiota in PCOS. In order to provide basis for prevention and treatment of PCOS based on gut microbiota.

## 1 INTRODUCTION

Polycystic ovary syndrome (PCOS) is a refractory female endocrine disease with complex aetiology and basic characteristics of persistent anovulation, insulin resistance (IR) and hyperandrogenism.^1^ As one of the modern refractory reproductive diseases, the incidence rate of PCOS is rising.^2^ The female irregular menstruation, infertility, obesity, and so forth caused by PCOS have become hot issues affecting family harmony and social development.^3^ The aetiology of PCOS is complex. In recent years, many scholars have found that its pathogenesis was related to the imbalance of gut microbiota.^4^, ^5^ It was found that the gut microbiota of PCOS patients changed. The imbalance of gut microbiota led to the occurrence and development of PCOS.^6^ This article reviews the relationship between PCOS metabolic abnormalities and gut microbiota, and discusses its possible mechanism and new treatment direction.

## 2 OVERVIEW OF GUT MICROBIOTA

Intestinal microecology is composed of gut microbiota and its host environment. Gut microbiota disorder will lead to various diseases.^7^, ^8^ The interaction between gut microbiota and environmental conditions can not only promote human metabolism and immune response, but also improve gut structure.^9^ Gut microbiota participates in human metabolism, immunity, endocrine and other functions through two‐way interaction with the external environment, thus affecting the physiological metabolism and pathological process, and is closely related to the production of various endocrine metabolic diseases (such as PCOS, diabetes, etc.).^10^ In 2012, Trema et al.^11^ found that dietary induction can result in gut microbiota structure change, intestinal barrier dysfunction, and endotoxemia, leading to chronic inflammation, IR, and hyperandrogenism in the body. The gut microbiota is also rich in various biological enzymes, which participate in the digestion and synthesis of various metabolic products to participate in host metabolism.^12^ At present, the interactions between host and gut derived metabolites, such as short‐chain fatty acid (SCFA), lipopolysaccharide (LPS), bile acid (BA), have been extensively studied. SCFA, branched‐chain amino acids (BCCA) and BA could regulate intestinal barrier integrity.^13^ The metabolic products of intestinal bacteria are disordered during regulation, which leads to the ‘leakage’ of LPS, thus disturbing immune system, insulin signal transduction, glucose metabolism and intestinal microbiota.^14^

## 3 CHANGES IN GUT MICROBIOTA IN PATIENTS WITH PCOS

PCOS is a complex endocrine metabolic disorder, and gut microbiota participates in various metabolic activities.^15^ The change of gut microbiota in patients with PCOS has been studied.^16^ A previous study demonstrated that the relative abundance of *Parabacteroides distasonis*, *Escherichia coli* and *Bacteroides fragilis* increased markedly in PCOS patients.^17^ Another study found that α diversity and β diversity of PCOS patients decreased compared to healthy controls. And *Porphyromonas* spp., *Bacteroides coprophilus*, *Faecalibacterium prausnitzii* increased significantly in patients with PCOS than the healthy women.^18^ At present, research on the relationship between PCOS and gut microbiota is mostly focused on PCOS patients who meet the Rotterdam diagnostic criteria, and there is a lack of classification research for different subtypes of PCOS patients. A recent study compared the gut microbiota between obese women with PCOS and without PCOS. The results showed that the richness and diversity of gut microbiota decreased in obese PCOS women. *Lachnoclostridium*, *Coprococcus*_*2* and *Tyzzerela* 4 were the major genera of obese PCOS patients.^19^ The changes of gut microbiota in PCOS patients could affect the occurrence and development of PCOS by changing intestinal permeability, synthetic metabolites and other pathways. Qi et al.^9^ transplanted the faeces of healthy or PCOS women to mice by oral lavage. The number of IR, ovarian cyst like follicles, testosterone and luteinizing hormone levels in the mice transplanted with faeces samples of PCOS patients increased PCOS like performance. It can be seen that gut microbiota plays a unique role in the occurrence of PCOS.

## 4 THE ROLE OF GUT MICROBIOTA IN THE PATHOGENESIS OF PCOS

### 4.1 Endotoxemia promotes chronic inflammation in PCOS

Studies demonstrated that various intestinal metabolites including SCFA, branched‐chain amino acid (BCAA), LPS and BA in host metabolism, could affect PCOS.^13^ Bacteroides and *E*. *coli* in human intestine belong to *Gram‐negative* bacteria. LPS is an important component of the cell wall of *Gram‐negative* bacteria. Studies demonstrated that the relative abundance of *E*. *coli* and *B*. *fragilis* increased markedly in PCOS patients.^17^ The destruction of the intestinal barrier and the increase in permeability promote the release of LPS, leading to the progression of clinical features of PCOS such as IR, HA, and abnormal follicular development. Through LBP, CD14 and MD‐2, LPS can be recognized by TLR4, which activates a wide range of cell signalling pathways and recruits downstream adaptor molecules such as TNF‐α and IL‐6, which subsequently activates the inflammatory response.^20^ Studies have shown that two groups of mice fed a normal and high fat diet for 1 month. The mice fed a high fat diet became obese and showed signs of IR. The blood LPS concentration increased 2–3 times than the control group. After infection of LPS for 4 weeks, the control group became obese and produced IR.^21^

### 4.2 Sex hormone levels affect the structure of gut microbiota

PCOS is a disease driven by high levels of androgens and low levels of oestrogen. Kelley et al.^22^ used trazole to induce the decline of gut microbiota diversity in Kaohsiung PCOS mouse model. The β‐glucuronic acid secreted by microorganisms can metabolize oestrogen from the binding form.^23^ The reduction of intestinal microbiota diversity will reduce the activity of β‐glucuronic acid, leading to the de binding of oestrogen and phytoestrogen, and the reduction of oestrogen in the circulation, thus reducing the active form of oestrogen receptor, which may lead to an increase in the risk of metabolic diseases.^24^ Guo et al.^25^ found that the levels of testosterone and androstenedione in the PCOS model rats induced by letrozole were significantly higher than those in the control group (*p* < 0.05). Prevotella changes with changes in testosterone and androstenedione levels. After faecal microbiota transplantation (FMT) or lactobacillus treatment, the levels of testosterone and androstenedione in PCOS model rats were significantly reduced (*p* < 0.05). The results of this study indicate a close relationship between gut microbiota and hyperandrogenism. Research has found that an increase in the numbers of Campylobacter, Desulfobacteria, and Bacteroidetes can lead to an increase in testosterone levels, while an increase in the numbers of Proteobacteria, Campylobacter, and Actinobacteria can help reduce testosterone levels.^19^

### 4.3 Branched‐chain amino acid affects insulin resistance

IR is the major clinical endocrine characteristics of PCOS patients.^26^ Excess insulin can promote the secretion of androgen by the ovary and adrenal gland and inhibit the processing and synthesis of sex hormone‐binding globulin by the liver, increase the relative content of free testosterone in the circulation, thus interfering with follicular development. The overall gut microbiota structure of the IR population has undergone significant changes. BCAA can trigger IR by activating mTOR, protein S6 kinase 1 (S6K1) and other kinases.^27^ It has been confirmed that the serum BCAA level of IR individuals increased. BCAA is a potentially harmful microbial regulatory metabolite. Intestinal microbiota can synthesize BCAA: leucine, isoleucine and valine, and gut microbiota can affect insulin sensitivity through BCAA.^28^ Pedersen et al.^29^ simulated the metabolic state of obese or long‐term high‐fat diet PCOS patients using a mouse model. Mice fed a high‐fat diet for 2 weeks exhibited higher BCAA. After 3 weeks of feeding, they exhibited varying degrees of IR. The possible mechanism is that BCAA metabolic disorder may exacerbate IR by altering glucose metabolism, resulting in the occurrence of PCOS.

### 4.4 The content of SCFA affects insulin resistance occurrence

SCFA is a product of anaerobic fermentation and degradation of carbohydrates in human intestinal tissue, with reduced levels in PCOS patients.^30^ A recent study demonstrated that several SCFA‐producing bacteria decreased significantly in patients with PCOS, including *Butyricimonas*, *Blautia*, *Coprococcus*, and *F*. *prausnitzii*.^31^ These bacteria are regarded as SCFA producers and have beneficial effects on hosts. *F*. *prausnitzii* exhibits anti‐inflammatory activity and maintains mucosal immune homeostasis.^32^ *Coprococcus* is a member of the *Anaerococcus* genus, the *Firmicutes* phylum, and the *Trichospiridae* family. It is an important genus of bacteria in the intestine and one of the important producers of butyric acid. Faecal cocci can be used as microbial biomarkers to evaluate human gastrointestinal health, and *Coprococcus* bacteria may help suppress immune responses and reduce the severity of inflammatory reactions.^33^ The decrease in SCFA levels can induce PCOS by disrupting intestinal barrier integrity.^34^ SCFA is closely related to blood glucose regulation, inflammatory response and sex hormone levels, and plays a key role in increasing intestinal barrier, regulating energy intake, and regulating immunity. Zhang et al.^35^ found that the contents of acetate, propionate and butyrate in the intestines of PCOS patients were significantly reduced by about 30% to 66%. After 10 weeks of probiotics treatment for PCOS patients, the abundance of lactobacilli in the intestines of PCOS patients were significantly increased, and the level of SCFA in the intestines was also significantly increased, promoting insulin secretion. Hong et al.^36^ supplemented some high‐fat diet mice with butyrate in their high‐fat diet research, effectively preventing the development of IR and obesity in mice. Other research found increasing dietary fibre and supplementing butyrate can reduce the occurrence of obesity and increase insulin sensitivity.^37^ The incidence of IR in women with PCOS ranges from 25% to 70%.^38^

### 4.5 The role of bile acid on PCOS

The close relationship between gut microbiota and bile acids is gradually being discovered. The gut microbiota can regulate the metabolism and transformation of bile acids, which have antibacterial effects and can affect the composition, structure and function of the gut microbiota. The interaction between bile acids and gut microbiota is involved in the occurrence and development of PCOS. There are literature reports that compared with the healthy control group, there is a significant increase in the faeces of PCOS patients with common *Bifidobacterium lactis*.^39^ Most gram‐positive bacteria, like *Ruminococcus* and *Clostridium*, and some gram‐negative bacteria can metabolize bile acids.^40^ Qi et al.^41^ found in animal experiments that healthy mice transplanted with faecal microbiota from women with PCOS or fed with bifidobacteria exhibited typical features of PCOS, including IR, estrous cycle disorder, and abnormal hormone levels. In clinical experimental studies, they further found that the abundance of *Pseudomonas aeruginosa* in PCOS patients significantly increased, leading to a significant decrease in the levels of glycine cholic acid deoxycholic acid (GDCA) and TUDCA in PCOS patients. After administering exogenous GDCA and TUDCA to PCOS mice, the phenotype of PCOS can be improved by upregulating the expression of interleukin‐22 (IL‐22).^41^

## 5 GUT MICROBIOTA AND PCOS TREATMENT

### 5.1 Faecal microbiota transplantation

FMT is a research hotspot in recent years.^42^, ^43^ FMT could directly and rapidly change the composition of the new host's gut microbiota and treating diseases.^44^ The change of gut microbiota results in *Clostridium difficile* infection.^45^ FMT is a way to help patients establish a new gut microbiota homeostasis by injecting faecal suspension from healthy donors into the intestines of recipient patients. FMT can be a valid support in treating obesity, multiple sclerosis, diabetes, PCOS, bacterial vaginosis, endometriosis and other diseases.^46^, ^47^ Guo et al.^25^ administered PCOS model rats with faecal supernatant of normal rats for 2 weeks, found that the levels of estradiol and estrone were significantly increased, androgen production were significantly reduced (*p* < 0.05), and the ovarian function and estrous cycle of PCOS model rats were significantly improved. Compared with the lactobacillus transplantation group, the FMT group can better reduce the androgen level of rats and improve the estrous cycle. The results showed that both FMT and lactobacilli transplantation could regulate gut microbiota by reducing the number of Pullorum and increasing the number of lactobacilli and Clostridium, so as to achieve the goal of treating PCOS. Furthermore, a recent study showed that FMT and curcumin combination is potential methods for PCOS.^48^ Although experiments have shown that FMT is an effective method for treating PCOS, it is still in the animal experimental research stage and further research is needed to be applied in clinical practice.

### 5.2 Probiotics and prebiotics

Studies have found that probiotics can regulate gut microbiota, treat metabolic diseases, and restore gut microbiota.^49^ *Lactobacillus* and *bifidobacteria* are the most commonly used probiotics at present. They can stimulate the natural immunity, maintain the stability of the normal gut microbiota of the human body, and play an important role in protecting human health. Animal experiments have proved that PCOS can be treated by supplementing probiotics to regulate gut microbiota.^25^ He et al.^50^ found through 16S rRNA sequencing that lactobacilli were related to the level of sex hormones, and plant lactobacilli could improve the intestinal microecology of PCOS model rats, play the role of protecting ovaries, reducing testosterone levels, restoring luteinizing hormone and follicle‐stimulating hormone levels, thereby alleviating the symptoms of PCOS. The study shows that compared with the vitamin D combined with placebo group, after 12 weeks of intervention with vitamin D combined with probiotics in patients with PCOS, the Beck Depression Inventory scores of patients were significantly lower, the general health questionnaire scores were significantly higher, and the serum total testosterone level was significantly lower (*p* < 0.05).^51^

Fructooligosaccharides, inulin, galactose and lactulose are common prebiotics, which can maintain the health by regulating the composition of microorganisms. Xue et al.^52^ divided 40 mice into four groups, with 10 mice in each group. In addition to the control group, the other three groups established PCOS models and were intervened with corresponding drugs for 21 days. The body weight, testosterone, plasma LPS, interleukin (IL)‐6 and IL‐17A in the inulin group were significantly lower. The results of sequencing analysis of gut microbiota showed that, compared with the model group, the number of bifidobacteria in the inulin group increased significantly. The study showed that the intervention of yoghourt supplemented with inulin in PCOS model mice induced by dehydroepiandrosterone for 24 days could increase the relative abundance of lactobacilli and bifidobacteria, reduce the body mass of mice (*p* < 0.05), and improve the estrous cycle and ovarian morphology.^53^ α‐lactalbumin (α‐LA), a component of lactose synthase, is a whey protein in milk. Recent studies demonstrated that α‐LA exhibited prebiotic activities and able to contribute to gut microbiota restoration.^54^ In particular, a recent review by Cardinale et al. reported its crucial role in the management of PCOS, highlighting its biological effects regarding the ability to increase serotonin levels (reduced in PCOS women) and to stimulate the proliferation of positive microbial populations, as *Bifodobacteria* and *Lactobacilli*.^55^ Another study in murine model of obesity by Boscaini et al., revealed the improvement of gut microbiota composition after α‐LA enriched diet.^56^ In addition, scientific evidence reported also the possibility for α‐LA to improve micronutrient intestinal absorption. Indeed, a recent article published by Kamenov et al., reported that adding α‐LA to the assumption of inositol–that are natural molecules effective in PCOS endocrine and metabolic recovering, strongly improves reproductive and metabolic outcomes.^57^ These studies indicated that α‐LA can be used as a new attractive molecule in the management of PCOS.

### 5.3 Lifestyle interventions

PCOS affects various physiological stages, age, medical needs and clinical manifestations of women, which determines that personalized treatment should be adopted for PCOS. Many studies recommend lifestyle intervention as the methods for PCOS treatment, and dietary changes can rapidly restore the gut microbiota.^58^ The imbalance of gut microbiota can lead to chronic low‐grade inflammation and metabolic disorder, aggravating the symptoms of PCOS.^59^ In addition to fat, other ingredients in the diet structure also have a regulatory effect on gut microbiota, in which plant protein, whole wheat and wheat bran and dietary polyphenols can increase the relative abundance of intestinal probiotics, while animal protein, glucose, fructose and sucrose have the opposite effect.^60^, ^61^ Therefore, in addition to medication and surgical therapy, the dietary structure of PCOS patients should also receive reasonable intervention. Common programs include: increase the proportion of unsaturated fat acid in daily diet, and improve PCOS symptoms by regulating the sex hormone microbiota inflammation axis.^62^ Encourage patients to increase their intake of water‐soluble dietary fibre, such as fruits and seaweed, and increase the SCFA of gut microbiota metabolites to protect the intestinal mucosa.^63^ It is recommended that patients reduce carbon and water intake, use the ketogenic diet (KD) mode, reduce the relative abundance of *Brautzia* and *Eubacterium halcyniae* in obesity core bacteria, and play a key role in weight loss.^64^ A previous study found that feeding flaxseed oil to PCOS model rats can improve the estrous cycle and ovarian function of rats, reduce blood lipids and IR, and regulate gut microbiota.^65^ Exercise can affect gut microbiota, and appropriate exercise can contribute to human health. An experiment was conducted to compare the effects of 6 weeks of exercise on the gut microbiota of mice. Compared with the sedentary group, the exercise group had a higher abundance of *Bacillota*, while the abundance of *Bacteroides* and *Proctor* decreased (*p* < 0.05).^66^ Although many previous studies have found that improving lifestyle can treat or reduce the risk of PCOS,^67^, ^68^ the mechanism of diet and exercise on gut microbiota affecting PCOS is not completely clear, which is worth further exploration.

## 6 CONCLUSIONS

In conclusion, there is a complex and close interaction between PCOS and gut microbiota. The relationship between the pathogenesis and pathophysiological processes of PCOS and the structure and function of gut microbiota needs further research. A deep understanding of the relationship between gut microbiota and metabolic abnormalities in PCOS provides new ideas for the prevention and treatment of PCOS and metabolic diseases based on gut microbiota as a target. Each individual has a unique set of gut microbiota, and the concept of healthy gut microbiota requires quantitative indicators. Further collection and analysis of large‐scale data are needed. The regulation of gut microbiota in PCOS patients provides new goals and choices for personalized treatment.

## AUTHOR CONTRIBUTIONS

**Min Zhou:** Conceptualization (equal); investigation (equal); writing – original draft (equal). **Jing Yu:** Investigation (equal); methodology (equal); software (equal). **Xuewei Li:** Conceptualization (equal); investigation (equal); software (equal). **Zheng Ruan:** Investigation (equal); methodology (equal); resources (equal). **Shaohui Yu:** Conceptualization (equal); investigation (equal); methodology (equal); writing – review and editing (equal).

## CONFLICT OF INTEREST STATEMENT

The authors have no relevant financial or non‐financial interests to disclose.

## CONSENT FOR PUBLICATION

All authors agree to publish in Journal of Cellular and Molecular Medicine.

## Data Availability

The data that support the findings of this study are available from the corresponding author upon reasonable request.
